# Mobilome Analysis of *Achromobacter* spp. Isolates from Chronic and Occasional Lung Infection in Cystic Fibrosis Patients

**DOI:** 10.3390/microorganisms9010130

**Published:** 2021-01-08

**Authors:** Laura Veschetti, Angela Sandri, Cristina Patuzzo, Paola Melotti, Giovanni Malerba, Maria M. Lleò

**Affiliations:** 1 Department of Neurosciences, Biomedicine and Movement Sciences, University of Verona, 37134 Verona, Italy; laura.veschetti@univr.it (L.V.); cristina.patuzzo@univr.it (C.P.); giovanni.malerba@univr.it (G.M.); 2 Department of Diagnostics and Public Health, Microbiology Section, University of Verona, 37134 Verona, Italy; angela.sandri@univr.it; 3 Cystic Fibrosis Center, Azienda Ospedaliera Universitaria Integrata Verona, 37126 Verona, Italy; paola.melotti@aovr.veneto.it

**Keywords:** mobilome, *Achromobacter*, lung infection, cystic fibrosis, horizontal gene transfer (HGT), prophages, integrative and conjugative elements (ICEs), insertion sequences (ISs), integrative and mobilizable elements (IMEs), antimicrobial resistance genes, virulence genes

## Abstract

*Achromobacter* spp. is an opportunistic pathogen that can cause lung infections in patients with cystic fibrosis (CF). Although a variety of mobile genetic elements (MGEs) carrying antimicrobial resistance genes have been identified in clinical isolates, little is known about the contribution of *Achromobacter* spp. mobilome to its pathogenicity. To provide new insights, we performed bioinformatic analyses of 54 whole genome sequences and investigated the presence of phages, insertion sequences (ISs), and integrative and conjugative elements (ICEs). Most of the detected phages were previously described in other pathogens and carried type II toxin-antitoxin systems as well as other pathogenic genes. Interestingly, the partial sequence of phage Bcep176 was found in all the analyzed *Achromobacter xylosoxidans* genome sequences, suggesting the integration of this phage in an ancestor strain. A wide variety of IS was also identified either inside of or in proximity to pathogenicity islands. Finally, ICEs carrying pathogenic genes were found to be widespread among our isolates and seemed to be involved in transfer events within the CF lung. These results highlight the contribution of MGEs to the pathogenicity of *Achromobacter* species, their potential to become antimicrobial targets, and the need for further studies to better elucidate their clinical impact.

## 1. Introduction

Horizontal gene transfer (HGT) is the transfer of genetic elements among organisms by means other than vertical transmission [1,2]. HGT is a well-described mechanism that has been increasingly studied due to its significant role in the rapid dissemination of genetic elements between bacteria [3,4,5,6,7,8,9]. Mobile genetic elements (MGEs) are segments of DNA that encode proteins mediating their own movement within intracellular mobility or HGT phenomena [10]. MGEs include a wide variety of different elements that can be horizontally transmitted in three ways: transformation, transduction, or conjugation.

Transformation is the process by which bacteria take up foreign DNA from the local environment [11], while transduction consists of a DNA transfer mediated by phages, also called bacteriophages [10]. These are bacterial viruses that can contain into their capsid elements of host DNA, which can be transferred to a new host and be inherited, thus allowing the host to express traits carried by phages [12]. Finally, conjugation consists of the transfer of DNA through cell-to-cell contact and requires independently replicating genetic elements such as conjugative plasmids and integrative and conjugative elements (ICEs) [7,13,14]. ICEs can integrate into and excise from genomes using integrase, circularize, replicate, and then transfer via conjugation [13,14,15]. Another type of MGE that can horizontally transfer through conjugation is integrative mobilizable elements (IMEs), which encode an integrase and circularize like ICEs but have to exploit the conjugative machinery of co-resident ICEs or conjugative plasmids [16]. Another type of MGEs is represented by insertion sequences (ISs), which are small DNA segments that are able to move themselves to new genomic locations within a single cell. ISs are often present in multiple copies in the same genome and can thus facilitate homologous recombination events [17].

MGEs have an important role in antimicrobial resistance and virulence [18,19,20,21] as well as in bacterial adaptation. Indeed, they encode genes related to these mechanisms and can enable their host to synthesize products that affect the fitness of co-infecting pathogens, like bacteriocins [22,23], or confer antibiotic resistance [24]. In particular, a strong link between ICEs and the dissemination of antibiotic resistance genes has been demonstrated [13]. It is thus clear that MGEs and HGT events contribute to the pathogenic potential of microbes [19,25,26]. Another mechanism favoring bacterial persistence is the ability to genetically adapt during chronic infection through the accumulation of pathoadaptive mutations [27]. This phenomenon can be accelerated due to mutations in the DNA mismatch repair system (MMR), giving rise to hypermutation events and to clonal diversification within the host [28].

Although MGEs have been detected in the great majority of prokaryotic organisms, the scale and importance of HGT events is still not clear for less characterized bacterial species such as *Achromobacter* spp., an opportunistic pathogen that can cause lung infections in patients with cystic fibrosis (CF). Even though chronic colonization has been associated with a decline in respiratory function, increased frequency of exacerbations, and lung inflammation [25,26,29,30], literature regarding *Achromobacter* mobilome and its contribution to pathogenicity is still scarce. Nonetheless, a rich variety of mobile genetic elements carrying resistance genes in addition to its natural multidrug resistance have been identified in clinical isolates [31].

To better understand the role and impact of MGE on *Achromobacter* pathogenicity potential, we performed whole genome sequencing of 54 clinical isolates and investigated the presence and content of phages, ISs, ICEs, and IMEs through bioinformatic analyses.

## 2. Materials and Methods

### 2.1. Samples Collection and Identification

Fifty-four isolates were collected from the sputum samples of 26 patients followed at the CF Center of Verona and were identified as *Achromobacter* spp. by MALDI-TOF-MS (bioMerieux Marcy-l’Étoile, France). Additional information on patients, type of infection (chronic, occasional), and sampling timeline was reported in Table S1. Informed consent was obtained according to projects CRCFC-CEPPO026 and CRCFC-CEPPO031 approved by the local Ethical Committee.

According to the European Consensus criteria (ECC), infection was defined as chronic when at least three positive cultures over ≥6 months were obtained with at least a 1-month interval between the samples [32]. Sputum samples were collected approximately every 3 months for microbiological analysis. The classification of occasional and chronic infection was assessed using the information regarding all the *Achromobacter* isolates identified between 2013 and 2018. A minimum of 1 isolate and a maximum of 6 isolates from each infected patient were stored and then used in this study. The collection activity was mainly performed in two time periods: 2014–15 and 2017–18. The average nucleotide identity (ANI) among all available *Achromobacter* spp. genomes (*n* = 142, NCBI RefSeq database, January 2020) and all sequenced isolates were calculated in order to verify the correct species classification of the reference and isolates genomes. Isolates were considered to belong to the same species when ANI ≥ 95%, whereas isolates with ANI < 95% with all available *Achromobacter* spp. genomes were considered as new genogroups (NG) [33]. Genotypic relatedness among longitudinal isolates was verified by checking core genome similarities obtained using the Harvest-OSX64-v1.1.2 suite [34].

### 2.2. Genome Sequencing

All the isolates underwent whole genome sequencing at the Technological Platform Centre of the University of Verona. Sequences were submitted to the NCBI SRA database with project number PRJEB40979. Genomic DNA was extracted using the QIAamp DNA Blood Mini Kit (Qiagen, Milan, Italy) and its quality was assessed using NanoDrop 2000 (Thermo Fisher Scientific, Wilmington, DE, USA) and a Fragment Analyzer System (Agilent Technologies, Santa Clara, CA, USA). Libraries were prepared using the KAPA PCR-free kit (Roche Sequencing Solutions, Pleasanton, CA, USA) and sequenced on a NextSeq500 Illumina platform (Illumina, Hayward, CA, USA) generating 150bp paired end reads with a mean read yield of 10978104 and a mean coverage of 190X. Read quality controls, de novo assembly, and genome annotation were performed similarly to our previous work [35]. Details on the sequencing and de novo assembly are available in Table S2.

### 2.3. Mobilome Analysis

Phage Search Tool Enhanced Release (PHASTER) [36] was used in order to identify and annotate the prophage sequences based on similarity. Only phages classified as intact by the tool were included into the subsequent analyses. Phylogenetic analysis of Burkho Bcep176 phage was performed by extracting the phage sequence from each *Achromobacter xylosoxidans* isolate and using the Parsnp tool of the Harvest-OSX64-v1.1.2 suite with the options -c -r ! -C 1000 [34]. The tree file in Newick format was used as an input in iTOL [37] for visualization. The presence of ICEs, IMEs, and cis-mobilizable elements was ascertained using the online version of the ICEfinder tool based on the ICEberg [38]. Results were checked to ensure that no ICEs spanning two different contigs were called. Finally, the ISfinder tool [39] was used to evaluate the presence of ISs. The identified mobile genetic elements annotations were manually investigated to evaluate the gene content according to the literature, i.e., presence of antimicrobial resistance genes, virulence factors, and other pathogenic genes. Heatmaps were generated for results visualization purposes using the pheatmap R package v1.0.8.

## 3. Results

Fifty-four *Achromobacter* spp. clinical isolates were longitudinally collected over 5 years from sputum samples of 26 patients followed at the CF Center of Verona (Italy). Among them, 17 presented chronic lung infection while nine were occasionally colonized. Genomic analysis identified five *Achromobacter* species: *Achromobacter aegrifaciens* (7%, *n* = 4), *Achromobacter dolens* (7%, *n* = 4), *Achromobacter insolitus* (6%, *n* = 3), *Achromobacter insuavis* (13%, *n* = 7), and *A. xylosoxidans* (67%, *n* = 36). Interestingly, four strains isolated from two patients showed an average nucleotide identity of <95% against all the other analyzed genomes, suggesting they likely belonged to *Achromobacter* species with no reference genomes available yet. In this study, we refer to them as new genogroups (NG).

### 3.1. Phage Analysis

**Figure 1:**
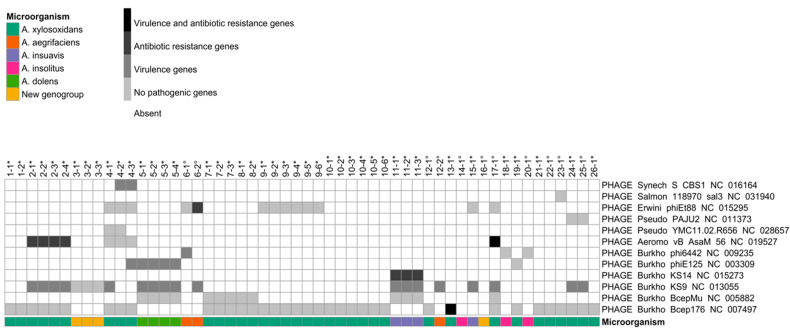
Phage analysis. The heatmap shows the presence of phages and the category of pathogenic genes that they carry (grey scale). The columns represent patients and isolates identification numbers. * indicates chronic infection isolates; ° indicates occasional infection isolates. Additional information regarding the microorganism species is represented in the annotation row (color scale) at the bottom.

Phage analysis (Figure 1) identified the presence of 12 bacteriophages that were first described in the following six host genera: *Burkholderia*, *Pseudomonas*, *Aeromonas*, *Erwinia*, *Salmonella,* and *Synechococcus*. Interestingly, the most represented ones are *Burkholderia* (six phages) and *Pseudomonas* (two phages), two of the major CF pathogens. In particular, Burkho Bcep176, and Burkho KS9 phages were identified in the isolates of a high number of patients (65% and 42%, respectively). While some phages were identified in different *Achromobacter* species, others were found to be specific for *A. xylosoxidans,* such as Burkho Bcep176, Aeromo vB AsaM, Pseudo YMC, Pseudo PAJU2, Salmon 118970, and Synech S CBS1. In particular, the tail sequence of the first one was present in all *A. xylosoxidans* isolates. Among the other species, only *A. insuavis* isolates presented Burkho KS14. Except for few cases, phage presence did not seem to be correlated to the type of infection (chronic or occasional). In chronic patients presenting clonal longitudinal isolates, the type and number of phages was consistent over time except for one case (patient 4). Moreover, in a patient with occasional infections, we observed variations between two longitudinal isolates belonging to the same clonetype. These two cases suggest that phage gain or loss might have happened within the CF lung environment.

Investigating the pathogenic genes carried by the identified phages, we observed that only Burkho Bcep176 and Aeromo vB AsaM carry both virulence and antibiotic resistance genes, while four phages (Burkho BcepMu, Pseudo YMC, Pseudo PAJU2, and Salmon 118970) carried none. Among the phages carrying pathogenic genes, the RelE/ParE and HicA/B type II toxin/antitoxin (TA) systems were identified (Table S3). These systems are involved in MGEs stability, biofilm formation, stress responses, and antibiotic persistence. Interestingly, in a Pseudo PAJU2 phage, we detected the presence of the l*exA* gene, which is involved in the repression of the SOS response to DNA damage, thus enhancing the mutation rate while preventing apoptosis.

### 3.2. Insertion Sequences (ISs) Analysis

**Figure 2:**
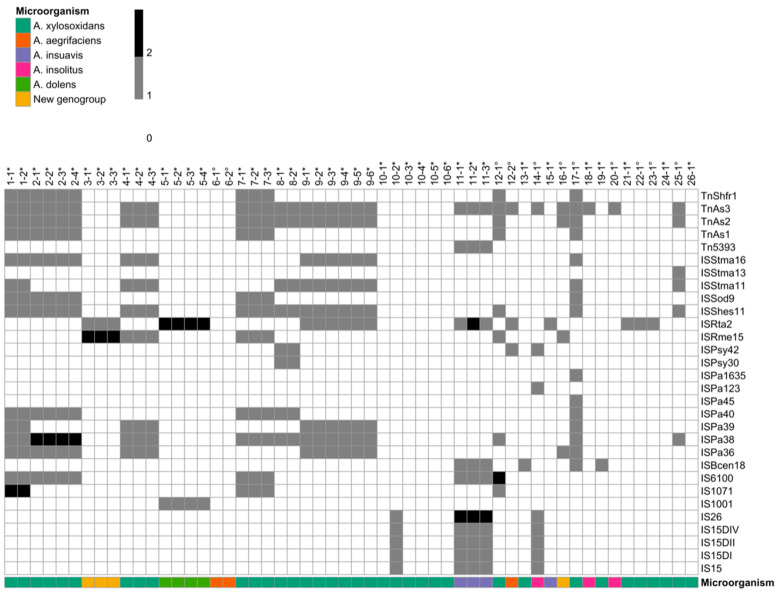
Insertion sequences (ISs) analysis. The heatmap shows the presence and number (grey scale) of ISs. The columns represent patients and isolates identification numbers. * indicates chronic infection isolates; ° indicates occasional infection isolates. Additional information regarding the microorganism species is represented in the annotation row (color scale) at the bottom.

The ISs analysis results (Figure 2) showed the presence of ISs from a wide variety of microorganisms, including opportunistic human pathogens such as *Burkholderia cepacia* complex, * Pseudomonas aeruginosa*, *Stenotrophomonas maltophilia*, *Ralstonia* spp., *Sphingomonas paucimobilis*, and *Bordetella parapertussis*, as well as environmental species like *Shewanella* spp., *Salmonella* spp., and *Pseudomonas syringae.* The majority of *A. xylosoxidans* and *A. insuavis* isolates carried more than two types of ISs simultaneously, while the other *Achromobacter* species harbored a maximum of two ISs types. Interestingly, ISs related to plasmid maintenance and rearrangement were found in nine isolates (all isolates from patients 1, 7, 8, 14, and isolate 12–1), indicating the possible presence of plasmids in these strains. Variations in ISs among clonal longitudinal strains were only observed in two chronic patients (10 and 11).

### 3.3. Integrative and Conjugative Elements (ICEs) and Integrative and Mobilizable Elements (IMEs) Analysis

**Figure 3:**
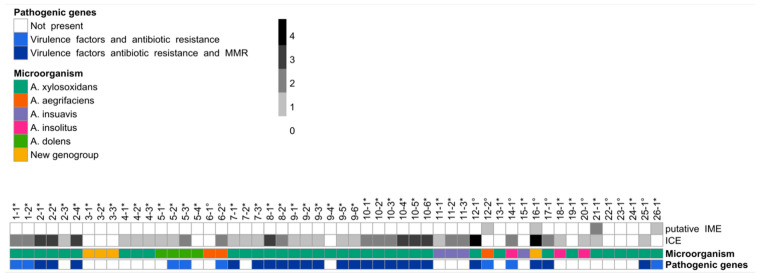
Integrative and conjugative elements (ICEs) and integrative and mobilizable elements (IMEs) analysis. The heatmap shows the presence and number (grey scale) of ICEs and IMEs. The columns represent patients and isolates identification numbers. * indicates chronic infection isolates; ° indicates occasional infection isolates. Additional information regarding the microorganism species and pathogenic genes carried by mobile elements is represented in the annotation row (color scale) at the bottom.

The presence of ICEs was widespread among our isolates (74%), while IMEs were only found in four strains (Figure 3). Interestingly, ICEs carrying a combination of virulence factors, antibiotic resistance genes, and mismatch repair (MMR) genes were only detected in *A. xylosoxidans*, while no pathogenic genes were identified in *A. insuavis* ICEs. Among the antibiotic resistance genes carried by ICEs, we found genes related to penicillin, beta-lactams, tetracycline, bleomycin, sulfonamide, novobiocin, and deoxycholate resistance in addition to antibiotic efflux systems. Various virulence factors were also identified (Table S4), including genes involved in a Type 4 Secretion System (T4SS), Type 2 Secretion System (T2SS), hemolysin, flagella, pilus, and proteases. Moreover, we frequently observed the presence of LysR-type transcriptional regulators, which control diverse set of genes, including those involved in virulence, metabolism, quorum sensing, and motility. Among MMR genes, *radC* was found in a high number (58%) of *A. xylosoxidans* isolates. Within longitudinal isolates, we observed variations in the presence and pathogenic content of ICEs over time, indicating the possible exchange of these elements within the CF lungs.

## 4. Discussion

*Achromobacter* spp. is an opportunistic pathogen that can cause lung infections in CF patients. Although chronic colonization has been associated with a decline in respiratory function, increased frequency of exacerbations, and lung inflammation, little is known about its pathogenic mechanisms. In particular, virulence features related to its ability to colonize chronically, or only occasionally, the lungs of CF patients are largely unknown. Infections are usually complicated by the innate and acquired multidrug resistance carried by these microorganisms. Moreover, a rich variety of mobile genetic elements carrying resistance genes have been identified in clinical isolates, such as plasmids, IS26, IS440, and class I and class II integrons [31,40]. To provide new insights about *Achromobacter* spp. mobilome and its role in virulence and antibiotic resistance, we performed genomic analyses of 54 clinical isolates mainly collected in 2014–15 and 2017–18. Patients carrying these isolates could be defined as chronically or occasionally infected based on their clinical microbiological history since 2013 and not restricted to the isolates that were included in this study. Although the two-period collection limits the continuous observation of MGEs evolution during these 5 years, it still provides longitudinal data that can support the understanding of MGEs role, particularly in poorly characterized microorganisms such as *Achromobacter* spp.

Well-represented MGEs in *Achromobacter* spp. are phages, which infect bacteria. They are the most abundant organisms in the biosphere [41], and they have been of interest to scientists as vectors of HGT and drivers of bacterial evolution In previous literature, *Sinorhizobium*, *Ralstonia*, *Pseudomonas*, and *Burkholderia* have been suggested to be the most likely to be involved in horizontal transfer with *Achromobacter* spp. [42], most likely because they share a similar GC content. MGEs genetic content can be functional even in evolutionarily distant genomic backgrounds, like in the case of transposable elements carrying “blurry” promoters [43]. Although phages previously reported in other environmental species have also been detected, the majority of identified phages in this study were first described in species of clinical interest. In particular, typical *Burkholderia* phages were the most frequently detected followed by typical *Pseudomonas* phages. Since both are among the main respiratory pathogens in CF patients, we can hypothesize that phages can be exchanged between bacteria through horizontal transfer during lung colonization. Moreover, the tail and host adsorption apparatus sequence of phage Bcep176 from *Burkholderia* was present in all *A. xylosoxidans* isolates. The ubiquity of this sequence within this specific species suggests an ancestral uptake of the phage, followed by the loss of its structural components and the consequent inability of further transfer. The phylogenetic analysis of this sequence confirmed a genetic relatedness (72% core genome similarity) among all *A. xylosoxidans* isolates (Figure S1). Additionally, its presence was also ascertained in two reference genomes (RefSeq accessions: NZ_LS483395.1 and NZ_LN831029.1), further supporting the hypothesis of integration of this phage in an ancestor strain of *A. xylosoxidans*.

The majority of detected phage sequences carried virulence or antibiotic resistance genes highlighting the importance of mobile genetic elements in the pathogenicity of *Achromobacter* spp. Interestingly, genes involved in MGE stability, biofilm formation, stress responses, and antibiotic persistence such as Type II TA systems were identified. These systems have been reported to occur more often in pathogenic bacteria than in nonpathogenic microorganisms and therefore have been correlated to bacterial pathogenicity [44,45,46,47,48,49,50]. Since they are present in bacteria but not in eukaryotic cells, they have also been evaluated as antimicrobial targets [51,52] that can lead to bacterial cell lethality both by the artificial activation of toxins and by targeting the TA operon promoter region. The further study and characterization of Type II TA systems in *Achromobacter* spp. could thus lead to the implementation of novel and alternative treatment regimens in CF therapy. In particular, the RelE/ParE and HicA/B type II TA systems have been identified in our collection of isolates. While the latter is induced by nutrient starvation and might provide a survival advantage in difficult conditions [53], the RelE/ParE system has been well characterized as a genetic element that promotes stable plasmid inheritance [54]. Although we cannot provide evidence of plasmids in these isolates due to a limit in our study design regarding genomic DNA extraction, we neither can exclude their presence in light of these results. Plasmid-related genes were also found within ISs carried by nine isolates. The presence of these systems within both phages and ISs encourages further studies regarding the presence and content of plasmids in *Achromobacter* spp.

IS elements are defined as small (<2.5 kbp) segments of DNA capable of inserting at multiple sites in the genome [55]. They show a simple genetic organization and usually cluster in islands within genomes. They participate in chromosome rearrangements and plasmid integration, and are involved in antibiotic resistance, gene acquisition, and many pathogenic and virulence functions. Indeed, IS sequences in 14 isolates were found to be inside or in close proximity of pathogenicity islands, including ICEs (Table S4). Previous studies also reported that the presence of ISs is frequently associated to antimicrobial resistance genes and to class I and II integrons [31,40].

Similar to phages, ISs from a wide variety of microorganisms were identified, particularly from the species of clinical interest including CF opportunistic pathogens such as *B. cepacia* complex, *P. aeruginosa*, and *S. maltophilia*. This further supported the possible occurrence of interspecies transfer of mobile genetic elements among CF pathogens. A high consistency of both phages and IS in terms of type and carried genes was observed among longitudinal strains, with the exception of a few isolates that showed gain or loss of these elements despite the brief time between isolation events. The transiency of these mobilome profiles might suggest the temporary presence of subpopulations rather than a longitudinal microevolution.

ICEs are self-transmissible mobile genetic elements that usually mediate the transfer of diverse properties to enable the host to better adapt to hostile conditions [14]. Although they have been scarcely reported in literature for *Achromobacter* species, ICEs were found to be widespread among our isolates. We observed the presence of genes involved in a variety of functions such as secretion, motility, quorum sensing, metabolism, MMR, and resistance to different classes of antimicrobial agents. Even though some IMEs have also been identified in our isolates, the distinction of IMEs and incomplete ICEs is still problematic for currently available bioinformatic tools when processing short-read draft assemblies.

While the other mobile genetic elements investigated in this study (phages and IS) showed high consistency in longitudinal isolates, we observed variations in the presence and pathogenic content of ICEs over time, indicating a frequent exchange of these elements within the CF lungs. This contribution to the genomic plasticity of *Achromobacter* isolates might play an important role in pathogenesis and adaptation during chronic infections.

The variety of MGEs identified in *Achromobacter* genomes and their diverse virulence and antibiotic resistance profiles confirm that *Achromobacter* spp. are a reservoir of HGT elements. Not only do they contribute to genomic plasticity, but some of these elements can even become a constitutive part of the bacterial genome, as supported by the presence of phage Bcep176 in all our *A. xylosoxidans* isolates as well as in the analyzed reference genomes. Our results highlight the contribution of mobile genetic elements to the pathogenic potential of *Achromobacter* species, the need for further studies to better elucidate their clinical impact, and their potential to become antimicrobial targets in treatment regimens.

## Data Availability

The genomic sequences analyzed in this study are openly available in NCBI SRA database within project number PRJEB40979.

## Supplementary Materials

The following are available online at https://www.mdpi.com/2076-2607/9/1/130/s1, Figure S1: Phylogenetic tree of phage Burkho Bcep176 tail sequence, Table S1: Sampling timeline, Table S2: Assembly details, Table S3: Phage content details, Table S4: ICE and IME content details.
