# Supplementary material for: Mobilome Analysis of Achromobacter spp. Isolates from Chronic and Occasional Lung Infection in Cystic Fibrosis Patients
Source: Microorganisms. 2021 Jan 8;9(1):130. doi: 10.3390/microorganisms9010130 (PMC7826576; doi:10.3390/microorganisms9010130)
Supplement: Supplementary file 1 [file microorganisms-09-00130-s001.zip › microorganisms-1033511-sl/00-Figure_S1.docx]

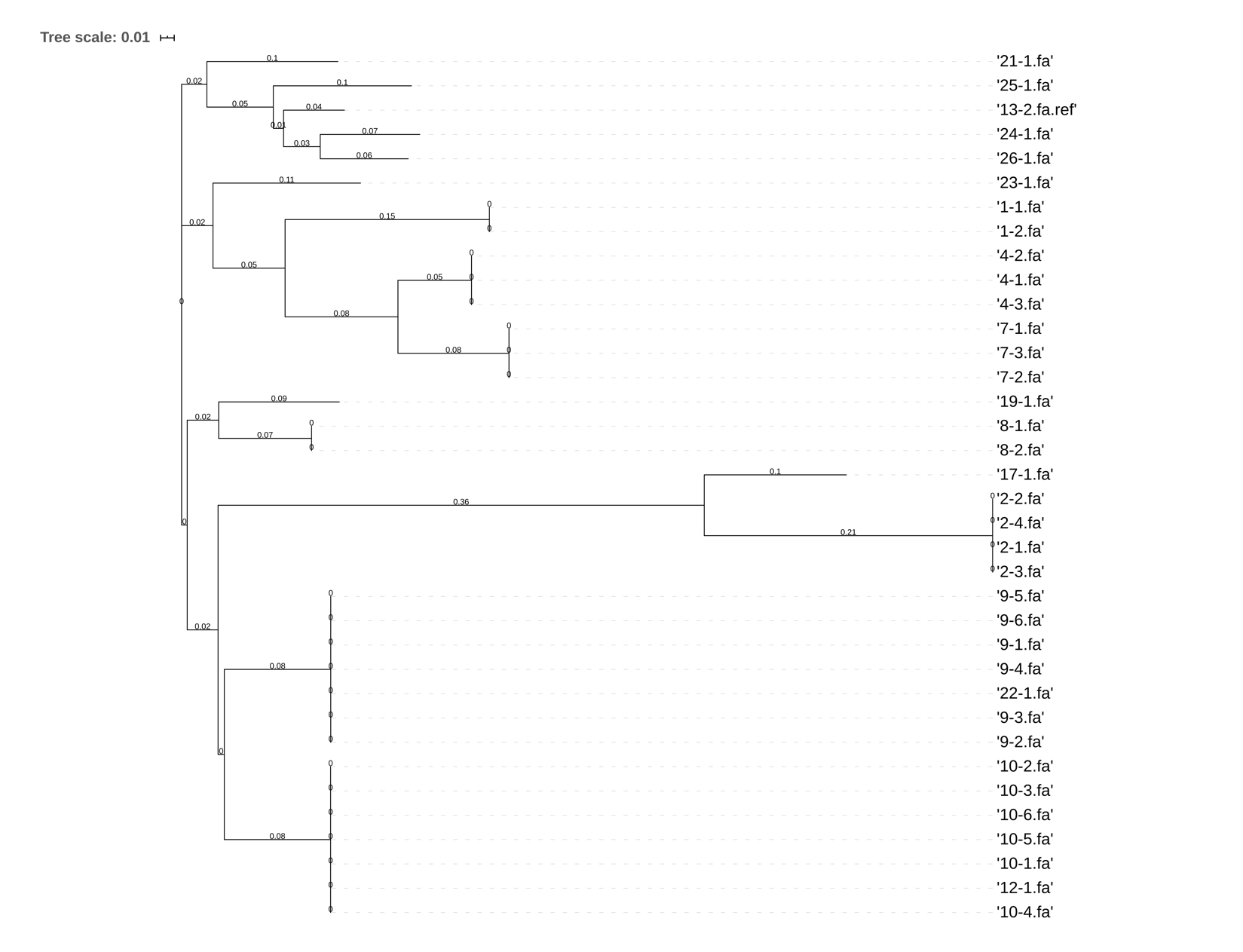


Figure S1: Phylogenetic tree of phage Burkho Bcep176 tail sequence. The scale is indicated in the upper left corner and phylogenetic distances rounded to the second decimal point are shown on tree branches.
